# Retraction Note to: Parecoxib inhibits esophageal squamous cell carcinoma progression via the PDK1–AKT pathway

**DOI:** 10.1186/s11658-022-00369-x

**Published:** 2022-08-15

**Authors:** Han-Ming Huang, Xiao-Yu Huang, Shao-Ping Wu, Can-Keng Chen, Xin-Hua He, Yong-Fa Zhang

**Affiliations:** 1 Department of Anesthesiology, Second Affiliated Hospital of Shantou University Medical College, Shantou, 515041 People’s Republic of China; 2 Department of Physiology, Shantou University Medical College, Shantou, 515041 People’s Republic of China

## Retraction to: Cellular & Molecular Biology Letters (2022) 27:28 https://doi.org/10.1186/s11658-022-00324-w

The Editor-in-Chief has retracted this article at the authors’ request. After publication, the authors became aware that the KYSE180 cells used in this study were cross-contaminated. Further checks by the publisher identified high similarity between the KYSE180 200 and 300 μM middle images. Additionally, the authors have stated that the number of injected cells for the in vivo tumor growth assay provided in the Materials and methods is incorrect.

All authors agree to this retraction.

